# Histone variant H2A.B-H2B dimers are spontaneously exchanged with canonical H2A-H2B in the nucleosome

**DOI:** 10.1038/s42003-021-01707-z

**Published:** 2021-02-12

**Authors:** Rina Hirano, Yasuhiro Arimura, Tomoya Kujirai, Mikihiro Shibata, Aya Okuda, Ken Morishima, Rintaro Inoue, Masaaki Sugiyama, Hitoshi Kurumizaka

**Affiliations:** 1 Laboratory of Chromatin Structure and Function, Institute for Quantitative Biosciences, The University of Tokyo, Bunkyo-ku, Tokyo Japan; 2 Department of Biological Sciences, Graduate School of Science, The University of Tokyo, Bunkyo-ku, Tokyo Japan; 3 Nano Life Science Institute (WPI-NanoLSI), Kanazawa University, Kakuma-machi, Kanazawa, Ishikawa Japan; 4 High-speed AFM for Biological Research Unit, Institute for Frontier Science Initiative, Kanazawa University, Kakuma-machi, Kanazawa, Ishikawa Japan; 5 Institute for Integrated Radiation and Nuclear Science (KURNS), Kyoto University, Sennan-gun, Osaka Japan

**Keywords:** Chromatin structure, Epigenetics, Biochemistry, Histone variants

## Abstract

H2A.B is an evolutionarily distant histone H2A variant that accumulates on DNA repair sites, DNA replication sites, and actively transcribing regions in genomes. In cells, H2A.B exchanges rapidly in chromatin, but the mechanism has remained enigmatic. In the present study, we found that the H2A.B-H2B dimer incorporated within the nucleosome exchanges with the canonical H2A-H2B dimer without assistance from additional factors, such as histone chaperones and nucleosome remodelers. High-speed atomic force microscopy revealed that the H2A.B nucleosome, but not the canonical H2A nucleosome, transiently forms an intermediate “open conformation”, in which two H2A.B-H2B dimers may be detached from the H3-H4 tetramer and bind to the DNA regions near the entry/exit sites. Mutational analyses revealed that the H2A.B C-terminal region is responsible for the adoption of the open conformation and the H2A.B-H2B exchange in the nucleosome. These findings provide mechanistic insights into the histone exchange of the H2A.B nucleosome.

## Abstract (web summary)

Hirano et al. show that the H2A.B-H2B histone dimer incorporated within the nucleosome exchanges with the canonical H2A-H2B dimer without assistance from histone chaperones or nucleosome remodelers. This study provides insights into how the H2A.B nucleosome exchanges its histones.

## Introduction

In eukaryotes, chromatin compacts genomic DNA for accommodation within the nucleus. The basic structural unit of chromatin is the nucleosome, which is composed of the nucleosome core particle (NCP) and linker DNAs. In the NCP, two histone H2A-H2B dimers associate with one histone H3-H4 tetramer, forming the histone octamer, and 145–147 base pairs of DNA are tightly bound to its basic surface^1,2^. Histones are stably incorporated into chromatin with a very slow exchange rate in living cells^3^, indicating that the NCP exists as a stable architecture. Therefore, the machineries managing genomic DNA functions, such as transcription, replication, repair, and recombination, must work on the DNA tightly wrapped within the NCP^4–6^. To relieve this NCP barrier in the genome, NCPs have versatile structures and dynamics^7–11^.

Histone variants are independently encoded by distinct genes^12,13^, and are considered to increase the variations in NCP structures and dynamics in chromatin^6,14–27^. The human testis-specific histone H3 variants, H3T and H3.5, form unstable NCPs, as compared to the canonical NCP^14,15^. CENP-A is a histone H3 variant that dictates the centromeric region of the chromosome, and forms an NCP structure with flexibly detached DNA ends^16–20^. In the NCPs containing histone H2A variants, H2A.Z.1 and H2A.Z.2, the overall NCP structures are equivalent to those of the canonical NCPs, but the local structures around the H2A.Z L1-loop regions are substantially different^21,22^.

**Fig. 1:**
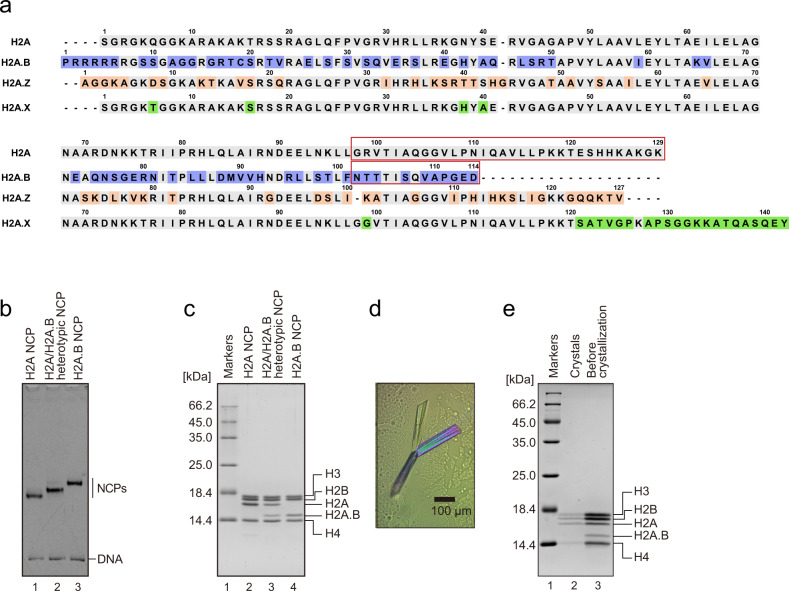
Crystallization of the H2A.B/H2A heterotypic NCP. **a** Sequence alignment of H2A, H2A.B, H2A.Z, and H2A.X. The specific residues of H2A.B, H2A.Z, and H2A.X, as compared with H2A, are colored blue, orange, and green, respectively. Dashes indicate gaps in the alignment. The C-terminal region of H2A (enclosed in the red square) is replaced with that of H2A.B (enclosed in the red square), in the chimeric protein shown in Fig. 5a and Supplementary Fig. 5. (a, b) The purified NCPs were analyzed by native-PAGE with ethidium bromide staining (**b**) and SDS-PAGE with Coomassie Brilliant Blue (CBB) staining (**c**). **d** The crystals obtained in the presence of the H2A.B/H2A heterotypic NCP (see “Methods”). **e** Histones in the crystals (lane 2) and in the solution of the H2A.B/H2A heterotypic NCP (lane 3) were analyzed by SDS-PAGE with CBB staining. The uncropped gel images are shown in Supplementary Fig. 9.

H2A.B (formerly named H2A.Bbd in human) is an evolutionarily distant histone H2A variant that conserves about 50% amino acid identity, as compared to the canonical H2A, and has short C-terminal tail^28^ (Fig. 1a). In mammalian cells, H2A.B reportedly accumulates on the transcription start sites^29–31^ and/or gene body regions of transcribing genes^31–34^. H2A.B also transiently assembles at DNA repair and replication sites^35,36^. These findings suggest that H2A.B may briefly form an NCP and protect the genomic DNA from endogenous and exogenous attacks, such as ionizing radiation or nucleases, when chromatin is re-configured after DNA transcription, repair, and replication. Indeed, H2A.B forms the NCP structure with flexible DNA ends in vitro^35,37–41^. The presence of flexible DNA ends in the H2A.B NCP is also supported by a molecular dynamics simulation study^42^. However, the biochemical properties of the H2A.B NCP have remained poorly understood.

In the present study, we found that the nucleosomal H2A.B-H2B dimer efficiently exchanges with the canonical H2A-H2B dimer, probably by forming an intermediate “open conformation” NCP structure. Interestingly, the H2A.B-specific C-terminal tail segment is important for the adoption of the open conformation and the H2A.B-H2B exchange in the NCP. These findings provide important insights into understanding the unusual behavior and function of H2A.B in cells.

## Results

### Nucleosomal H2A.B exchanges with canonical H2A without additional factors

H2A.B is a rapid-exchanging histone variant in cells^35,43^. To study the structural features of the H2A.B NCP, we reconstituted the NCP with recombinant human histones, H2A.B, H2B, H3, and H4, and obtained its crystals. However, the crystals of the H2A.B NCP generated poor X-ray diffraction data, probably due to the flexible nature of its DNA ends^35,37–41^. We previously reported that the crystal of the heterotypic NCP with the histone variant, CENP-A, which forms an NCP with flexible DNA ends, diffracted better than that of the homotypic NCP^19,44^. This fact led us to prepare the heterotypic NCP containing one each of H2A.B and H2A in the NCP, to improve the quality of the crystals (Fig. 1b, c). We then performed the X-ray crystallographic analysis (Fig. 1d). Surprisingly, the putative H2A.B/H2A heterotypic NCP lacked H2A.B and was formed with two canonical H2As (Supplementary Fig. 1 and Table 1), although the NCP sample before crystallization actually formed the heterotypic NCP containing both the H2A.B and H2A proteins (Fig. 1e). This implied that, during the crystallization processes, the nucleosomal H2A.B may be exchanged with the canonical H2A without assistance from additional factors.

**Table 1:** X-ray crystallography data collection and refinement statistics.

	NCP
**Data collection**	
Space group	*P*2_1_2_1_2_1_
Cell dimensions	
*a*, *b*, *c* (Å)	98.56, 107.71, 168.16
α, β, γ (°)	90.000, 90.000, 90.000
Resolution (Å)	50.00–2.6 (2.69–2.60)
*R*_merge_	9.5 (50.1)
*I* / σ*I*	69.4 (5.6)
Completeness (%)	98.5 (96.5)
CC_1/2_ in outer shell	0.747
Redundancy	5.3 (4.4)
**Refinement**	
Resolution (Å)	49.72–2.6
No. reflections	54914
*R*_work_ / *R*_free_	19.7/24.8
No. atoms	
Protein	5975
DNA	5967
Water	0
Ion	4
*B*-factors	
Protein	50.2
DNA	76.4
Water	–
Ion	82.8
R.m.s. deviations	
Bond lengths (Å)	0.010
Bond angles (°)	1.228

Values in parentheses are for highest-resolution shell.

### Nucleosomal H2A.B-H2B exchanges with free H2A-H2B in solution

**Fig. 2:**
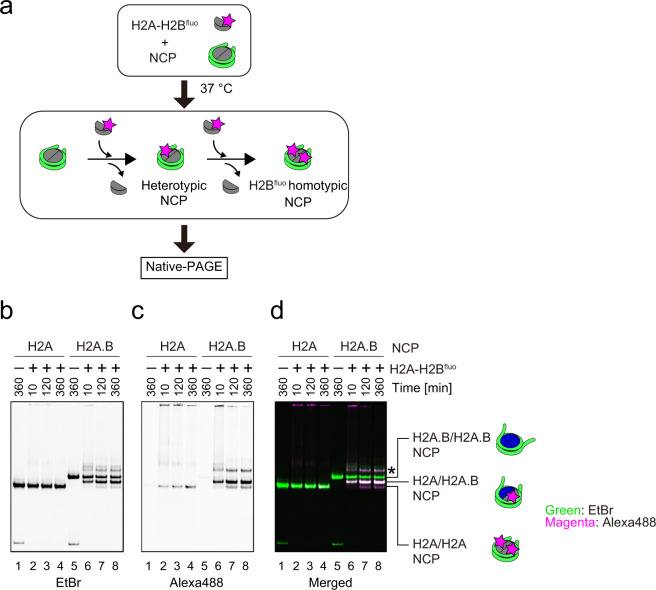
Histone exchange activity of the H2A.B NCP. **a** Schematic representation of the histone exchange assay. The NCP was incubated with the H2A-H2B dimer, in which the H2B protein was conjugated with the Alexa Fluor 488 fluorescent dye (H2A-H2B^fluo^ dimer). The samples were analyzed by native-PAGE. **b**–**d** Representative gel images of the histone exchange assay. The H2A NCP (1 µM) was incubated with the H2A-H2B^fluo^ dimer (0 µM: lane 1; 2 µM: lanes 2, 3, and 4) for 10 (lane 2), 120 (lane 3), and 360 (lanes 1 and 4) minutes. The H2A.B NCP (1 µM) was incubated with the H2A-H2B^fluo^ dimer (0 µM: lane 5; 2 µM: lanes 6, 7, and 8) for 10 (lane 6), 120 (lane 7), and 360 (lanes 5 and 8) minutes. After the incubation, the samples were analyzed by native-PAGE. The gels were visualized by ethidium bromide staining, or through the Alexa488 conjugated with the H2A-H2B dimer. **b** Ethidium bromide; **c** Alexa488 (H2A-H2B^fluo^); **d** Overlay of ethidium bromide (green) and Alexa-488 (magenta). The asterisk indicates the NCP-histone complexes. Reproducibility is confirmed by three independent experiments, and the results are presented in Supplementary Fig. 3. The uncropped gel images are shown in Supplementary Fig. 9.

We then performed the histone exchange assay to determine whether the nucleosomal H2A.B is actually exchanged with the canonical H2A, without additional factors. In this assay, the H2A.B NCP or the canonical NCP (H2A NCP) was incubated with the purified H2A-H2B dimer, and the resulting NCPs were analyzed by native polyacrylamide gel electrophoresis (PAGE). To confirm the incorporation of the H2A-H2B dimer exogenously added to the NCP, we used a fluorescently labeled H2A-H2B dimer (H2A-H2B^fluo^) as the exogenously added H2A-H2B dimer (Fig. 2a and Supplementary Fig. 2). Since the H2A.B NCP migrates more slowly in 6% native PAGE as compared to the canonical H2A NCP, the H2A-H2B^fluo^ dimer exchange can be detected by the migration change of the resulting NCPs, in addition to the fluorescence signal (Fig. 2b, Supplementary Fig. 3). As shown in Fig. 2b–d (lanes 5–8), the H2A-H2B^fluo^ dimer was substantially incorporated into the NCP, when the H2A.B NCP was incubated with the H2A-H2B^fluo^ dimer. In contrast, only a trace amount of the H2A-H2B^fluo^ dimer was incorporated into the H2A NCP by spontaneous histone exchange, when the H2A-H2B^fluo^ dimer was added to the reaction mixture (Fig. 2b–d, lanes 1–4). These results indicated that the H2A.B-H2B dimer in the NCP spontaneously exchanges with the canonical H2A-H2B dimer.

### The H2A.B NCP forms an open conformation

**Fig. 3:**
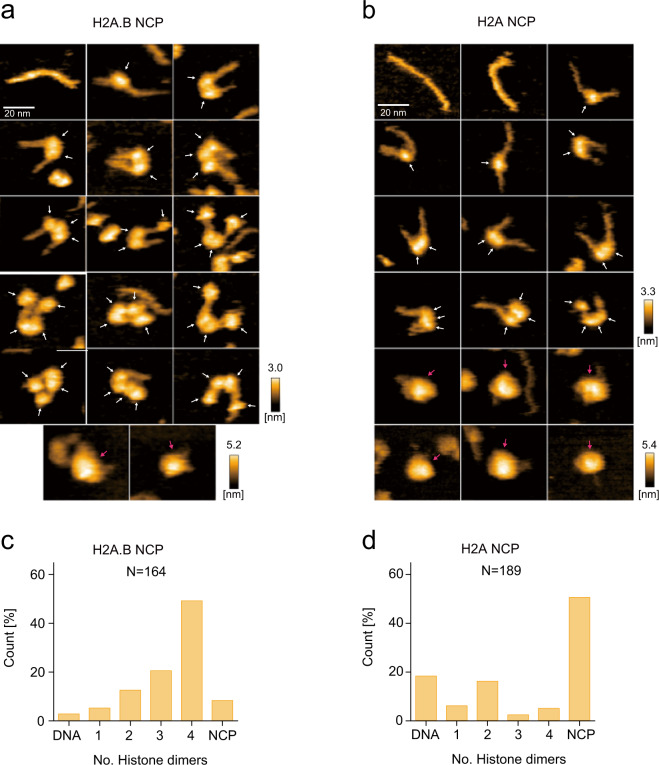
HS-AFM analyses of the NCPs containing H2A.B and H2A. **a**, **b** Initial HS-AFM images of the H2A.B NCP (**a**) and the H2A NCP (**b**). Individual particles are presented in the panels. White and magenta arrows indicate possible histone dimers and octamers, respectively. Scale bars are 20 nm. **c**, **d** The numbers of histone dimers in individual particles of the H2A.B NCP (**c**) and the H2A NCP (**d**) were counted and plotted. *N* indicates the total number of the counted particles. The source data for HS-AFM analysis are shown in Supplementary Data 1.

To understand the mechanism of H2A.B-H2B dimer exchange in the H2A.B NCP, we performed a high-speed atomic force microscopy (HS-AFM) analysis^45^. This method allows the visualization of the dynamic structural transition of the nucleosome^46–48^. We found that 49.4% of the H2A.B NCP existed as the open conformation, in which two small histone complexes (probably H2A.B-H2B dimers) are bound to the DNA and detached from the large histone complex (probably H3-H4 tetramer) at the initial stage of the HS-AFM analysis (Fig. 3a, c). In contrast, in the canonical H2A NCP, only a small proportion (5.3%) of the open conformation was observed at the initial stage (Fig. 3b, d).

**Fig. 4:**
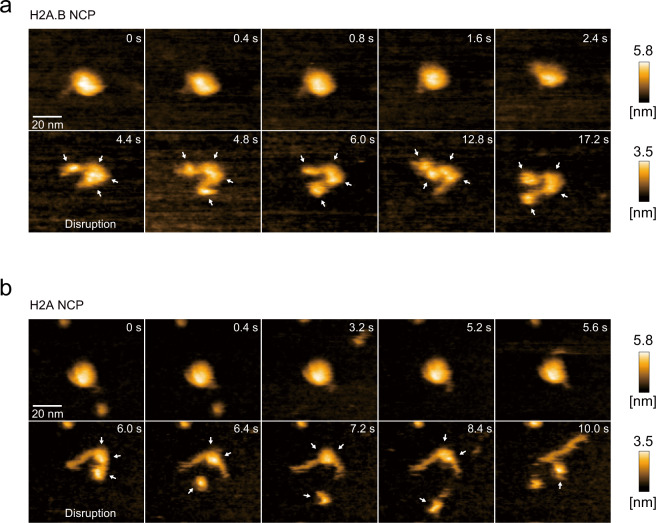
HS-AFM observations of the NCPs containing H2A.B and H2A. **a**, **b** Sequential HS-AFM images of the H2A.B NCP (**a**) and the H2A nucleosome (**b**). Representative NCPs with about 6 nm heights are shown, and the dissociation processes of the NCPs elicited by scratching with the HS-AFM probe were monitored. White arrows indicate possible histone dimers. Scale bars are 20 nm. Frame rate is 2.5 frames per second.

We selected NCPs with about 6 nm heights and monitored the NCP dissociation induced by scratching with the HS-AFM probe (Fig. 4). We found that the putative H2A.B-H2B dimers bound to the detached DNA region were continuously observed in the H2A.B NCP (Fig. 4a). This suggested that the H2A.B NCP was transformed into the open conformation (Fig. 4a and Supplementary movie S1). In contrast, in the H2A NCP, the putative H2A-H2B dimers were rapidly released from the DNA, and an obvious open conformation of the H2A NCP was rarely observed when the H2A-H2B dimer was released (Fig. 4b and Supplementary movie S2). The dwelling time of the H2A.B-H2B dimer on the nucleosomal DNA was quite long (average 24.6 s), as compared to that of the canonical H2A-H2B dimer (average 4.4 s), during the NCP disruption process (Supplementary Fig. 4). These results suggested that the H2A.B NCP, but not the H2A NCP, may dynamically adopt the open conformation.

### The H2A.B C-terminal region is responsible for the open conformation adoption and the H2A-H2B exchange activity

**Fig. 5:**
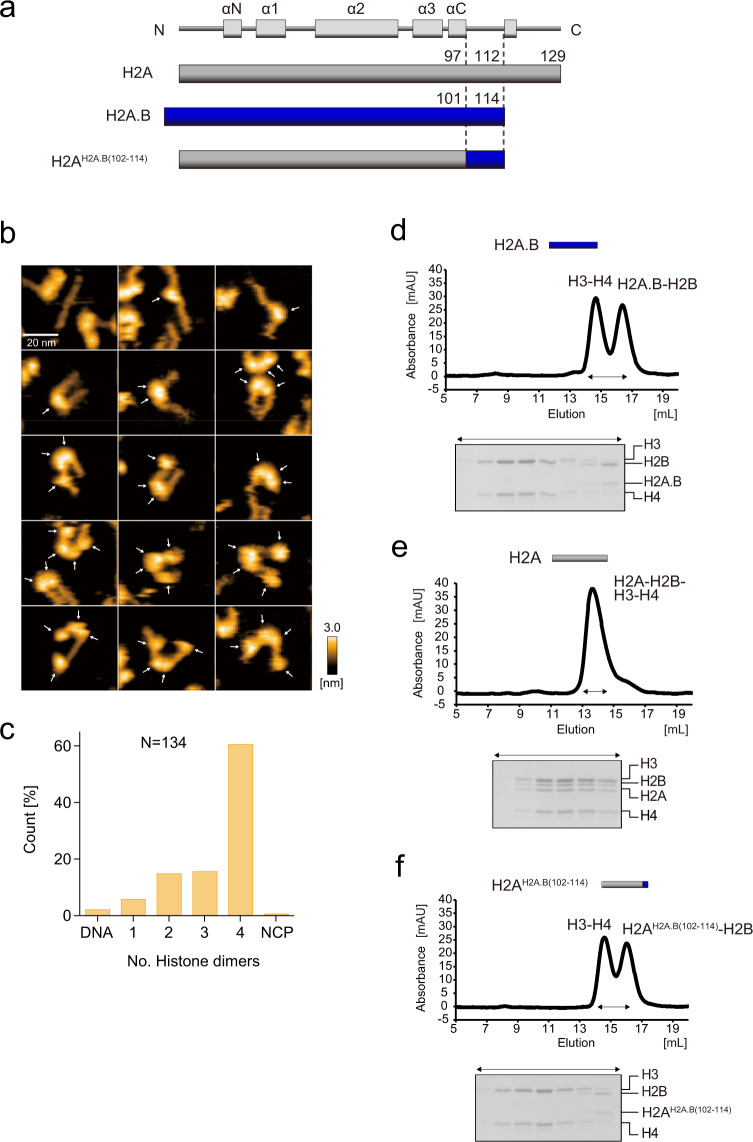
HS-AFM observation and histone octamer formation assay of the H2A^H2A.B(102–114)^ NCP. **a** Graphical representation of the chimeric protein used in the HS-AFM observations shown in (**b**, **c**) and the histone octamer formation assay shown in (**d**–**f**). **b** Initial HS-AFM images of the H2A^H2A.B(102–114)^ NCP. Individual particles are presented in the panels. White arrows indicate possible histone dimers. Scale bars are 20 nm. **c** The numbers of histone dimers in individual particles were counted and plotted. N indicates the total number of the counted particles. The source data for HS-AFM analysis are shown in Supplementary Data 1. **d**–**f** The H3-H4 tetramers were mixed with the H2A.B-H2B dimer (**d**), the H2A-H2B dimer (**e**), or the H2A^H2A.B(102–114)^-H2B dimer (**f**), and incubated in the presence of 2 M NaCl at 37 °C for 30 min. After the incubation, the samples were subjected to chromatography on a Superdex 200 10/300 GL column. Histone compositions of the peak fractions were analyzed by 18% SDS-PAGE with CBB staining. Reproducibility is confirmed by two independent experiments, and the results are presented in Supplementary Fig. 7. The uncropped gel images are shown in Supplementary Fig. 9.

The H2A.B variant is smaller than the canonical H2A, because of its shorter C-terminal tail region (Fig. 1a). In addition, the C-terminal amino acid sequence of H2A.B is not conserved among the H2A variants^28^ (Fig. 1a). We hypothesized that the C-terminal region of H2A.B may play a role in its specific characteristics, such as the adoption of the open conformation. To test this hypothesis, we prepared the canonical H2A mutant, H2A^H2A.B(102–114)^, in which the C-terminal region (amino acid residues 98–129) of canonical H2A is replaced with the corresponding H2A.B C-terminal region (amino acid residues 102–114) (Figs. 5a and 1a). The NCP containing the H2A^H2A.B(102–114)^ mutant was reconstituted (Supplementary Fig. 5). Consistent with our hypothesis, most of the H2A^H2A.B(102–114)^ NCP formed an open conformation similar to that of the H2A.B NCP detected by HS-AFM (Fig. 5b, c, Supplementary Fig. 6, and Supplementary movie S3).

We next performed a small-angle X-ray scattering (SAXS) analysis. In this method, the apparent NCP volume can be evaluated as the radius of gyration (*R*g). As shown in Table 2, the *R*g value of the H2A.B NCP was 56.5 ± 0.5 Å, which is substantially larger than that of the canonical NCP (44.9 ± 1.0 Å). Intriguingly, the *R*g value of the H2A^H2A.B(102–114)^ NCP was 52.9 ± 0.4 Å, which is also substantially larger than that of the canonical NCP (Table 2). These results supported the idea that the C-terminal region of H2A.B plays a role in forming the open conformation of the NCP in solution.

**Table 2:** SAXS analyses of the H2A, H2A.B, and H2A^H2A.B(102–114)^ NCPs.

NCP	*R*_g_ [Å]
H2A	44.9 ± 1.0
H2A.B	56.5 ± 0.5
H2A^H2A.B(102–114)^	52.9 ± 0.4

The detachment of the H2A.B-H2B dimers from the H3-H4 tetramer in the NCP may be important for generating the open conformation. The cryo-EM structure of the H2A.B NCP demonstrated that the H2A.B-H2B dimers associate with the H3-H4 tetramer in the NCP^41^. However, our gel filtration chromatography experiments revealed that, in the absence of DNA, the H2A.B-H2B dimer eluted separately from the H3-H4 tetramer, and did not form a histone octamer under conditions with 2 M NaCl (Fig. 5d, Supplementary Fig. 7a). In contrast, the canonical H2A-H2B dimers associate with an H3-H4 tetramer and form a histone octamer under the same experimental conditions (Fig. 5e, Supplementary Fig. 7b). These differences indicate that the association of the H2A.B-H2B dimer with the H3-H4 tetramer is weaker than that of the H2A-H2B dimer with the H3-H4 tetramer. This is perfectly consistent with the previous reports^37,41^. Interestingly, the H2A^H2A.B(102–114)^-H2B dimer, like the H2A.B-H2B dimer, eluted separately from the H3-H4 tetramer (Fig. 5f, Supplementary Fig. 7c). These results supported the hypothesis that the weak association between the H2A.B-H2B dimer and the H3-H4 tetramer in the NCP is mediated by the H2A.B C-terminal region, and may be required for the formation of the open conformation of the NCP.

**Fig. 6:**
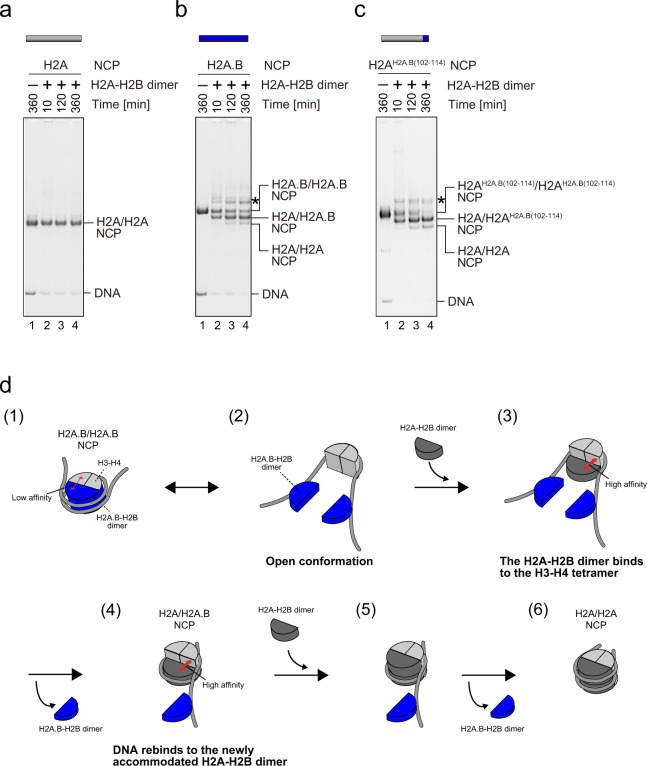
Histone exchange activity of the H2A^H2A.B(102–114)^ NCP. **a**–**c** Representative gel images of the histone exchange assays of the H2A (**a**), H2A.B (**b**), and H2A^H2A.B(102–114)^ (**c**) NCPs. The NCPs (1 µM) were incubated with the H2A-H2B dimer (0 µM: lane 1; 2 µM: lanes 2, 3, and 4) for 10 min (lane 2), 120 min (lane 3), and 360 min (lanes 1 and 4). After the incubation, the samples were analyzed by native-PAGE with ethidium bromide staining. The asterisk indicates the NCP-histone complexes. Reproducibility is confirmed by three independent experiments, and the results are presented in Supplementary Fig. 8. The uncropped gel images are shown in Supplementary Fig. 9. **d** Model showing the spontaneous exchange of the H2A.B-H2B dimer with the canonical H2A-H2B dimer in the H2A.B NCP. When the H2A.B NCP forms an open conformation (2), the H2A-H2B dimer (dark gray) preferably binds to the H3-H4 tetramer (light gray) because of its higher affinity to the H3-H4 tetramer (3). The DNA rebinds with the newly accommodated H2A-H2B dimer (4). Another H2A-H2B dimer then binds to the NCP (5), and the remaining H2A.B-H2B dimer (blue) dissociates from the DNA (6).

We finally tested whether the H2A.B C-terminal region functions in the H2A-H2B exchange. As expected, the exchange rates of the H2A^H2A.B(102–114)^-H2B dimers and the wild-type H2A.B-H2B dimers in the NCPs with the H2A-H2B dimers were similar (Fig. 6a–c, Supplementary Fig. 8). Therefore, we concluded that the H2A.B C-terminal region may enhance the H2A-H2B exchange in the NCP, through the adoption of the open conformation (Fig. 6d model).

## Discussion

H2A.B is a mysterious histone variant with quite interesting structural and physical characteristics^35,37–42,49–51^. Histone exchange is usually facilitated by factors, such as histone chaperones and nucleosome remodelers^5,52^. The nucleosomal H2A.B-H2B exchange is also reportedly promoted by the histone chaperone Nap1^53^. Surprisingly, we found that the H2A.B-H2B dimer incorporated into the NCP efficiently exchanges with the canonical H2A-H2B dimer, without assistance from additional factors (Figs. 1 and 2).

Previous studies demonstrated that H2A.B is incorporated into chromatin at replication and repair sites, and rapidly exchanged within several minutes in cells^35,43^. The exchangeable H2A.B variant may be utilized as a histone that temporarily protects the naked DNA regions emerging during DNA replication and repair. This rapid removal of the nucleosomal H2A.B may be mediated by the spontaneous H2A.B-H2B exchange with the canonical H2A-H2B. H2A.B also accumulates around the transcription start sites and/or gene bodies of transcribed genes^29–34^. The NCP containing the H2A.B-H2B dimers has a tendency to adopt the open conformation (Figs. 3 and 4), in which the nucleosomal DNA may become more accessible to DNA-interacting factors, such as transcription factors and RNA polymerase^54,55^. It will be intriguing to study the transcription efficiency on the nucleosome containing H2A.B.

H2A.B reportedly exists in spermatogenic cells and sperm^56^. During spermatogenesis, the chromatin architecture drastically changes and most of the nucleosomes are replaced by protamines in sperm^57^. The H2A.B-H2B exchange activity may be important to promote transitions of the chromatin architecture in the testis. A mouse histone H2A variant, H2A.L.2, reportedly functions in the histone replacement process by transition proteins and protamines^26^. Although mouse H2A.L.2 lacks a human homolog, like human H2A.B, it contains a shortened C-terminal region^58^. We determined that the H2A.B C-terminal region is responsible for the H2A.B-H2B exchange activity via the adoption of the open conformation (Figs. 5 and 6). In addition, both mouse H2A.L2 and human H2A.B are retained in sperm^56,57,59–61^. These similarities suggest that, in humans, H2A.B may serve as a counterpart to mouse H2A.L.2, which plays an essential role in spermatogenesis^26^.

In the present study, we found that the H2A.B NCP forms an open conformation, which may be an intermediate structure for the H2A.B-H2B exchange in the NCP (Figs. 3 and 4). In light of this finding, we propose a model for the nucleosomal H2A.B-H2B exchange (Fig. 6d). In this model, the H2A.B NCP (closed conformation) dynamically adopts the open conformation, which may facilitate access to the H3-H4 tetramer (Figs. 6d, (1) to (2)). A conformational change between the closed and open conformations may occur, because the association of the H2A.B-H2B dimer with the H3-H4 tetramer is substantially weaker, as compared to that between the H2A-H2B dimer and the H3-H4 tetramer^37,41^ (Fig. 5). This is consistent with the fact that the H2A docking domain (mapped to its C-terminal region), which interacts with the H3-H4 tetramer in the histone octamer, is not conserved in H2A.B^37,39^. In the canonical NCP, the H2A C-terminal residues (P109 and I111), which are not conserved in H2A.B, directly interact with the H3 residues (L48, I51, and Q55). In the open conformation, the H2A-H2B dimers may bind to the H3-H4 tetramer, because the H3-H4 surface becomes accessible in this conformation (Figs. 6d, (3)), and the H2A.B-H2B dimers could be evicted from the nucleosomal DNA (Figs. 6d, (4)). This model is consistent with the previous mutational analysis, in which the mutations of the histone H3 l51 and Q55 residues, located on the binding surface with the H2A C-terminal region in the NCP, enhanced the H2A-H2B exchange rate^62^.

The nucleosome has a stable architecture that often becomes an obstacle for gene functions, such as transcription, DNA replication, DNA recombination, and DNA repair. This negative effect of the nucleosome is utilized to regulate the genomic DNA functions, and may play a central role in the epigenetic regulation of genes in eukaryotes. Histone variants are considered to provide versatility in the nucleosome structures and physical properties, and play important roles in the epigenetic regulation of the genome^6–11^. The nucleosomal H2A-H2B exchange revealed in the present study is a quite unique characteristic specific for the H2A.B variant, and may play an important role in epigenetic regulation in mammals. Detailed structural studies of the open conformation of the H2A.B NCP are awaited.

## Methods

### Preparation of histones and histone mutants

The human histones, H2A, H2B, H2B(T122C), H3.1, H4, H2A.B, and H2A^H2A.B(102–114)^, were produced as recombinant proteins in *Escherichia coli* cells, and purified by the methods described previously^35,63^. Briefly, proteins were produced in *E. coli* BL21(DE3) cells as hexa-histidine (His_6_)-tagged proteins. The His_6_-tagged proteins were purified by Ni-NTA agarose (QIAGEN) chromatography under denaturing conditions. Except for H2A.B and H2A^H2A.B(102–114)^, the His_6_-tag was removed by cleavage with thrombin protease, and the histone proteins were further purified by MonoS cation exchange column chromatography. The His_6_-tags of H2A.B and H2A^H2A.B(102–114)^ were removed after the H2A.B-H2B or H2A^H2A.B(102–114)^-H2B dimer formation, as described below. The resulting proteins were desalted and lyophilized.

### Preparation of the histone complexes

The H2A-H2B, H2A-H2B(T122C), H2A.B-H2B, H2A^H2A.B(102–114)^-H2B dimers, and the H3.1-H4 tetramer were prepared as described previously^63^. The H2A^H2A.B(102–114)^-H2B dimer was purified by the same method used for the H2A.B-H2B dimer preparation. For the H2A-H2B dimer, the H2A-H2B(T122C) dimer, and the H3-H4 tetramer, the freeze-dried histones were mixed at equal molar ratio, and dissolved in 20 mM Tris-HCl (pH 7.5) buffer, containing 7 M guanidine-HCl and 20 mM 2-mercaptoethanol. The complexes were refolded by dialysis against refolding buffer, containing 10 mM Tris-HCl (pH 7.5), 2 M NaCl, 1 mM EDTA, and 5 mM 2-mercaptoethanol. The complexes were further purified by chromatography on a Superdex 200 gel filtration column (GE Healthcare).

The purified His_6_-tagged H2A.B or H2A^H2A.B(102–114)^ was mixed with H2B in 1:1 stoichiometry, and dissolved in 20 mM Tris-HCl (pH 7.5) buffer, containing 7 M guanidine-HCl and 20 mM 2-mercaptoethanol. The H2A.B-H2B and H2A^H2A.B(102–114)^ -H2B complexes were refolded by dialysis against refolding buffer, containing 10 mM Tris-HCl (pH 7.5), 2 M NaCl, 1 mM EDTA, and 5 mM 2-mercaptoethanol. After dialysis, the buffer was exchanged to refolding buffers containing 1 M, 0.5 M, and 0.1 M NaCl, in a stepwise manner. The His_6_-tag was removed by a treatment with thrombin protease (Wako), and the complex was further purified by chromatography on a Superdex 200 gel filtration column (GE Healthcare). Alexa488 labeling of the H2A-H2B (T122C) dimer was performed as described previously^64^. The purified H2A-H2B(T122C) complex was conjugated with Alexa Fluor 488 C5 Maleimide (invitrogen) in 10 mM Tris-HCl (pH 7.5) buffer, containing 2 M NaCl, 1 mM EDTA, and 1 mM TCEP. The reaction was stopped by adding 2-mercaptoethanol. The sample was then dialyzed against 10 mM Tris-HCl (pH 7.5) buffer, containing 2 M NaCl, 1 mM EDTA, and 5 mM 2-mercaptoethanol.

### Purification of the NCPs

The NCPs were prepared by the salt dialysis method with the palindromic 146 base-pair α-satellite DNA fragment, as described previously^1,65^. The DNA fragment containing one half of the α-satellite DNA fragment in pGEM-T Easy vector was amplified in the *E. coli* strain DH5α, and was excised from the plasmid DNA by *Eco*R V (Takara). The DNA fragment was then dephosphorylated by alkaline phosphatase (Takara), and was further cleaved by *Eco*R I. The DNA fragment was purified by DEAE-5PW anion-exchange column chromatography (TOSOH). The DNA fragment was self-ligated by T4 DNA ligase (NIPPON GENE), and the resulting DNA fragment was further purified by DEAE-5PW anion-exchange column chromatography (TOSOH).

The reconstituted NCPs were purified by non-denaturing gel electrophoresis, using a Prep Cell model 491 apparatus (Bio-Rad). For the H2A/H2A.B heterotypic NCP preparation, the 146 base-pair DNA, the H2A-H2B dimer, the H2A.B-H2B dimer, and the H3.1-H4 tetramer were mixed in a 1:0.85:2.55:1.7 molar ratio. The NCPs were reconstituted by the salt dialysis method. As a result, H2A/H2A, H2A/H2A.B, and H2A.B/H2A.B NCPs were reconstituted. The resulting three types of NCPs were separated by non-denaturing gel electrophoresis, using a Prep Cell model 491 apparatus (Bio-Rad), and the heterotypic NCP was selectively purified.

### Crystallization of the heterotypic NCP

The NCPs were dialyzed against 20 mM potassium cacodylate buffer (pH 6.0) containing 1 mM EDTA, for crystallization. The crystals were obtained by the hanging drop method. The NCP (1 µl, 2.33 mg/ml DNA concentration) was mixed with 1 µl of 20 mM potassium cacodylate buffer (pH 6.0), containing 50 mM KCl and 70–190 mM MnCl_2_. The mixture was equilibrated against 500 µl of reservoir solution, containing 20 mM potassium cacodylate (pH 6.0), 40 mM KCl, and 55–110 mM MnCl_2_. Crystals of the NCPs were obtained in 6–8 weeks. The crystals were cryoprotected by soaking in 19 mM potassium cacodylate buffer (pH 6.0), containing 5% trehalose, 30% polyethylene glycol 400, 38 mM KCl, and 86 mM MnCl_2_. The crystals were then flash-cooled in liquid nitrogen.

### Determination of the crystal structure

The X-ray diffraction data were collected at the beamline BL41XU (wavelength: 1.00000 Å) at SPring-8 (Harima, Japan). The diffraction data were scaled and processed using the HKL2000 program^66^. To prepare the search model for molecular replacement, the H2A atomic coordinates were removed from the human NCP structure (PDB ID: 5Y0C)^67^. The molecular replacement was performed with the PHASER program^68^. The atomic coordinates were refined using the PHENIX and Coot programs^69,70^.

### Histone exchange assay

The fluorescently labeled H2A-H2B dimer (2 μM) was mixed with the NCPs (1 μM), and the mixture was incubated at 37 °C for 10, 120, and 360 min in 14 mM Tris-HCl buffer (pH 7.5), containing 150 mM NaCl, 0.8 mM EDTA, 1.5 mM dithiothreitol, and 3 mM 2-mercaptoethanol. The resulting samples were subjected to 6% non-denaturing PAGE. The gel was stained with ethidium bromide, and the H2A-H2B dimer was visualized by the Alexa488 fluorescence.

For the exchange assay with H2A^H2A.B(102–114)^, the H2A-H2B dimer (2 μM) was mixed with the NCP (1 μM), and the mixture was incubated at 37 °C for 10, 120, and 360 min in 14 mM Tris-HCl buffer (pH 7.5), containing 150 mM NaCl, 0.8 mM EDTA, 1.5 mM dithiothreitol, and 3 mM 2-mercaptoethanol. The resulting samples were subjected to 6% native PAGE, and the gel was stained with ethidium bromide.

### HS-AFM observations

HS-AFM images of NCPs were obtained with our laboratory-build microscope, as described previously^71^. Briefly, HS-AFM was performed in the tapping mode. Deflections of the cantilever were detected by a two-segmented PIN photodiode, using an infrared laser (0.8 mW, 780 nm) focused through a ×60 objective lens (Nikon, CFI S Plan Fluor ELWD 60x) onto the back side of a cantilever (Olympus, BL-AC10DS-A2) covered with a gold coating. The free oscillation amplitude of the cantilever was ~1 nm, and the set-point amplitude was ~90% of the free amplitude for feedback control of HS-AFM. An amorphous carbon tip grown by electron beam deposition (EBD) was used as the AFM probe. For HS-AFM observations of NCPs, a mica surface was treated for 5 min with 50 μg/mL poly-L-lysine (mol. wt. 1000~5000, Sigma-Aldrich). HS-AFM observations were performed at room temperature (~25 ˚C), in a buffer consisting of 20 mM Tris-HCl (pH 7.5), 100 mM KCl, and 0.03% NP-40. HS-AFM observations of each NCP were performed at least three times to confirm the reproducibility. The imaging rate of HS-AFM is 2.5 frames per second for 100 × 80 nm^2^. HS-AFM images were collected using Igor Pro Ver. 8.0.4.2. (WaveMetrics). The images were analyzed using Igor Pro Ver. 8.0.4.2. (WaveMetrics) and ImageJ.

### SAXS analysis

SAXS was performed with a NANOPIX instrument (RIGAKU) at the Institute of Radiation and Nuclear Science, Kyoto University. To cover the wide *q*-range, we measured the sample with two sample-to-detector positions: 1330 mm for 0.007 –0.03 Å^−1^, and 300 mm for 0.03–0.8 Å^−1^, and then combined the measurements. After the standard procedures of transmission correction, buffer scattering subtraction, and conversion to an absolute scale with water scattering, we obtained the scattering profile of the NCPs. The NCP concentrations were 1.49 mg/mL, in 20 mM Tris-HCl (pH 7.5), 50 mM NaCl, and 1 mM dithiothreitol, and the temperature was kept at 20 °C.

We first examined the sample structure with the Guinier formula, which is established in the low *q*-range

$$(q) = \frac{{4\pi }}{\lambda }\sin \left( {\frac{\theta }{2}} \right),$$

(*λ* and *θ* are the X-ray wavelength and the scattering angle, respectively).

$$I\left( q \right) = I\left( 0 \right){\mathrm{exp}}\left( { - \frac{{R_g^2}}{3}q^2} \right),$$

where *I*(0) and *R*_*g*_ are the zero angle scattering intensity and gyration radius, respectively. *R*_*g*_ values were calculated with standard error. The observed SAXS intensity was corrected for background scattering, empty cell scattering, buffer scattering, and transmission factors, and subsequently converted to the absolute scale by SAngler (http://pfwww.kek.jp/saxs/SAngler.html). The Guinier analysis was performed with the linear least square method by Igor Pro (7.04).

### Gel filtration assay

The H3-H4 tetramer (2.75 nmol) was incubated with the H2A-H2B dimer (5.5 nmol), the H2A.B-H2B dimer (5.5 nmol), or the H2A^H2A.B(102–114)^-H2B dimer (5.5 nmol), in 100 μl of refolding buffer containing 2 M NaCl, at 37 °C for 30 min. After the incubation, 500 μl of refolding buffer containing 2 M NaCl was added to each sample mixture. The samples were then subjected to chromatography on a Superdex S200 10/300 gel filtration column (GE Healthcare). The peak fractions were analyzed by 18% SDS-PAGE with Coomassie Brilliant Blue staining.

### Statistics and reproducibility

For HS-AFM imaging experiments, the numbers of total counted particles are presented in Figs. 3c, d, 5c, and Supplementary Fig. 4b, c. The histone exchange assay was repeated three times. The octamer formation assay was repeated two times.

### Reporting summary

Further information on research design is available in the Nature Research Reporting Summary linked to this article.

## Supplementary information

Supplementary Information

Description of Additional Supplementary Files

Supplementary Video 1

Supplementary Video 2

Supplementary Video 3

Supplementary Data 1

Reporting Summary

## Data Availability

The uncropped images of gels are shown in Supplementary Fig. 9. The structural data of the NCP shown in Supplementary Fig. 1 have been deposited to the Protein Data Bank (PDB ID: 6V2K). The source data for HS-AFM analysis are shown in Supplementary Data 1. All other data are available from the authors upon reasonable request.
