# Supplementary material for: Histone variant H2A.B-H2B dimers are spontaneously exchanged with canonical H2A-H2B in the nucleosome
Source: Commun Biol. 2021 Feb 12;4:191. doi: 10.1038/s42003-021-01707-z (PMC7881002; doi:10.1038/s42003-021-01707-z)
Supplement: Supplementary file 1 — Supplementary Information [file 42003_2021_1707_MOESM1_ESM.pdf]

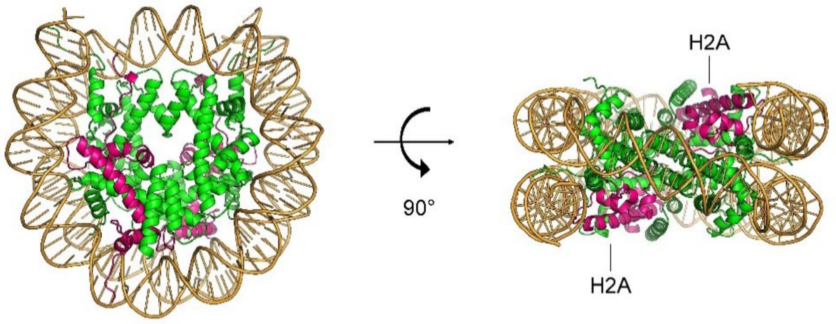


**Supplementary Figure 1. Crystal structure of the NCP obtained from the crystals shown in Figure 1c.**

The H2A molecules are colored pink, the H2B and H3 and H4 molecules are green, and the DNA is yellow.

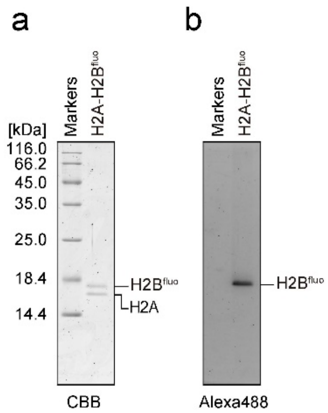


**Supplementary Figure 2. Preparation of H2A-H2B$^{fluo}$.**

(a, b) The purified H2A-H2B$^{fluo}$ dimer was analyzed by SDS-PAGE with CBB staining (a) or through H2A-H2B$^{fluo}$ (b).

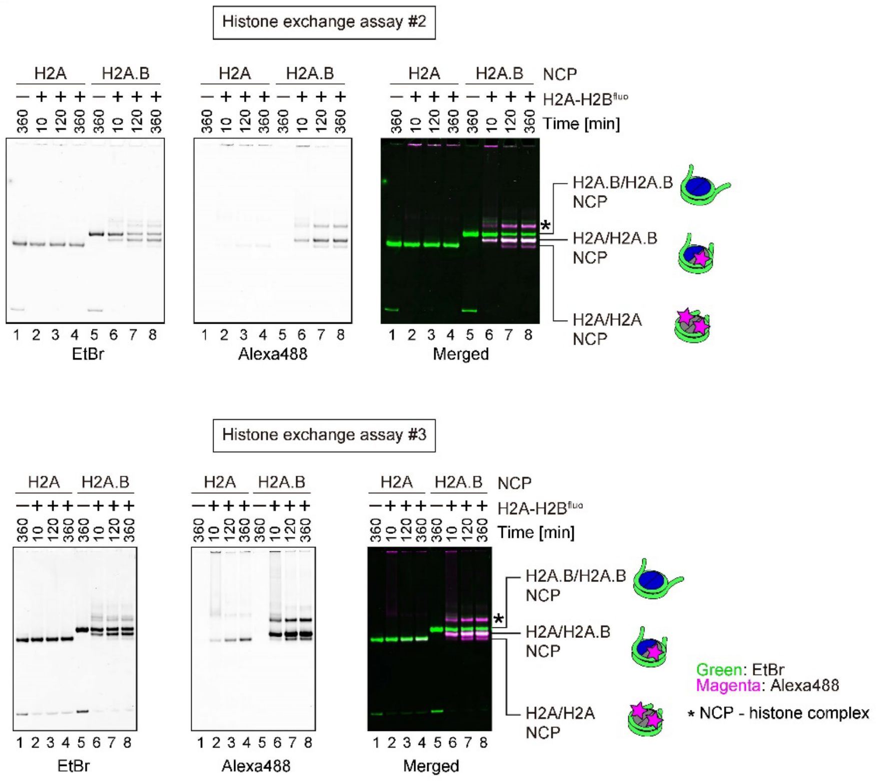


**Supplementary Figure 3. Replicated experiments of the histone exchange assay for the H2A.B NCP.**

Replicated experiments of the histone exchange assay shown in Figure 2b-d.

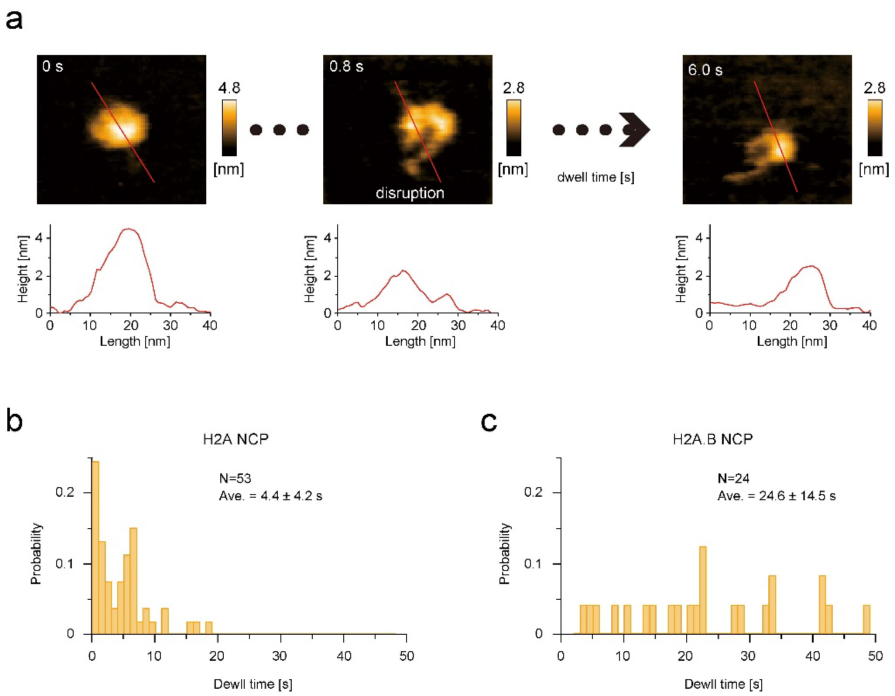


**Supplementary Figure 4. HS-AFM dwelling time analysis of the histone dimers retained in the NCPs.**

(a) Scheme for the dwelling time analysis. The sizes of the particles (height and width) are analyzed on the red line. (b, c) Plots of the dwelling times of histone dimers in the H2A.B NCP (b) and H2A NCP (c). N indicates the total number of the counted particles.

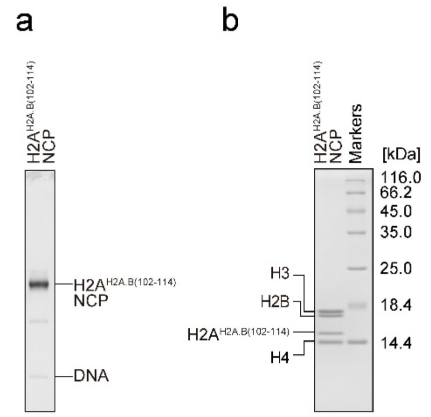


**Supplementary Figure 5. Preparation of the H2A$^{H2A.B(102-114)}$ NCP.**

(a, b) The purified NCP containing H2A$^{H2A.B(102-114)}$ was analyzed by native-PAGE with ethidium bromide staining (a) and SDS-PAGE with CBB staining (b).

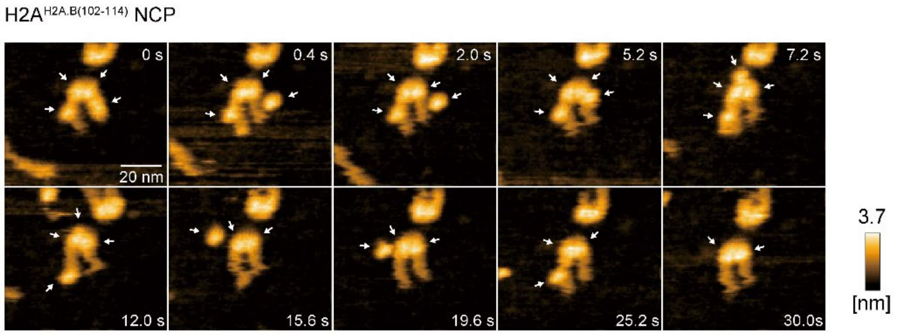


**Supplementary Figure 6. HS-AFM observations of the NCP containing H2A$^{\mathrm{H2A.B(102\text{-}114)}}$.**

Sequential HS-AFM images of the H2A$^{\mathrm{H2A.B(102\text{-}114)}}$ NCP. White arrows indicate histone dimers. Scale bars are 20 nm.

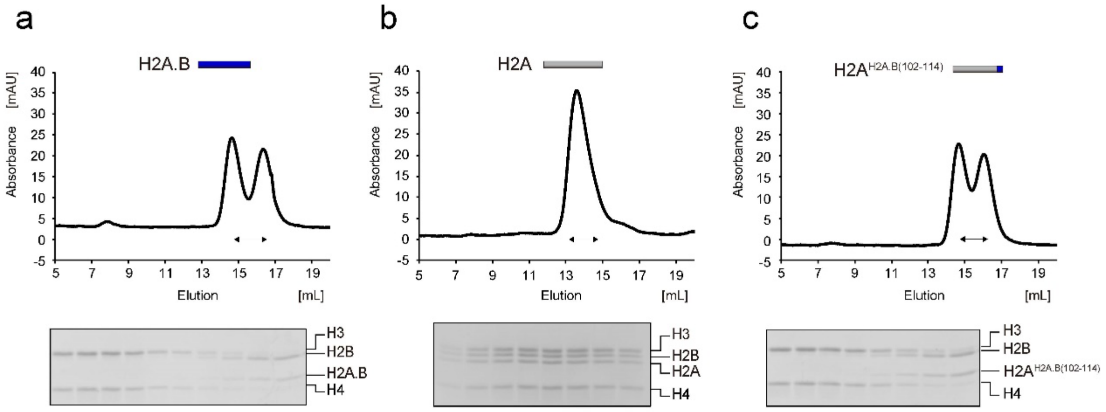


**Supplementary Figure 7. Replicated experiments of the octamer formation assay.**
(a-c) Replicated experiments of the octamer formation assay shown in Figure 5d-f.

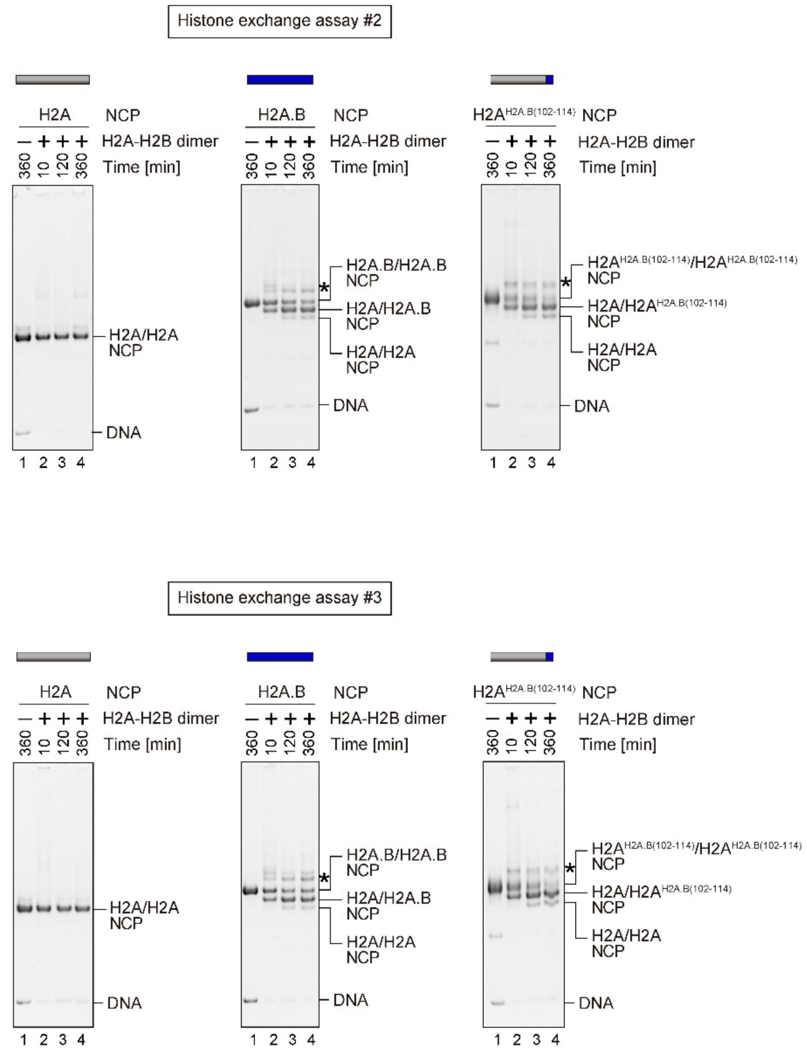


**Supplementary Figure 8. Replicated experiments of the histone exchange assay.** Replicated experiments of the histone exchange assay shown in Figure 6a-c.

Fig. 1b

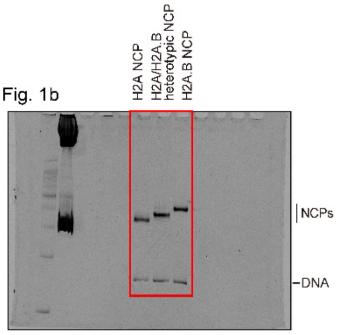


Fig. 1c

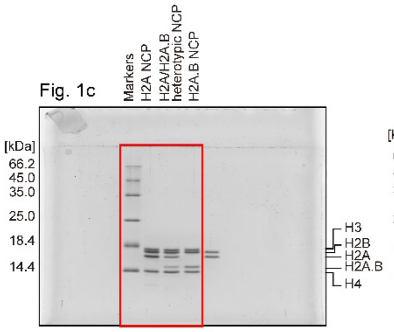


Fig. 1e

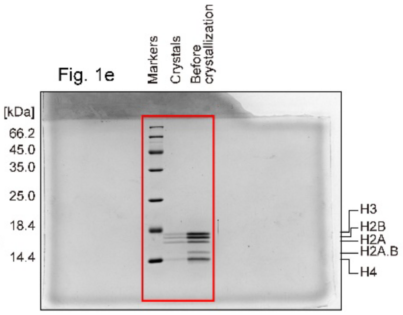


Fig. 2b

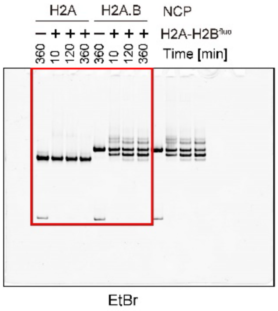


Fig. 2c

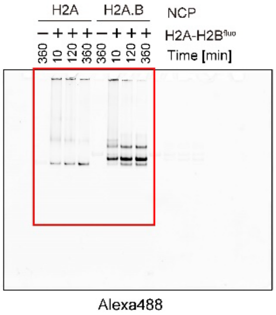


Fig. 2d

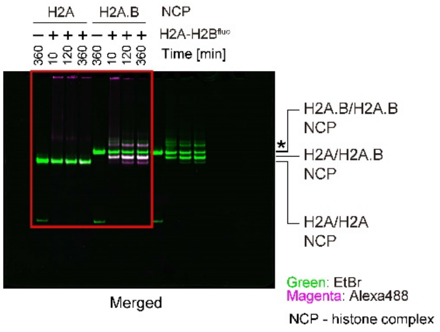


Fig. 5d

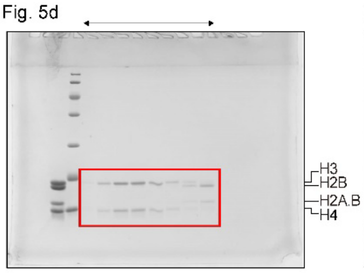


Fig. 5e

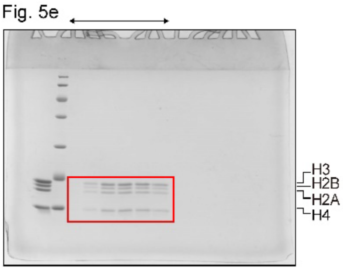


Fig. 5f

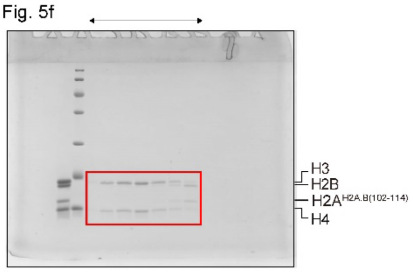


Fig. 6a Fig. 6b

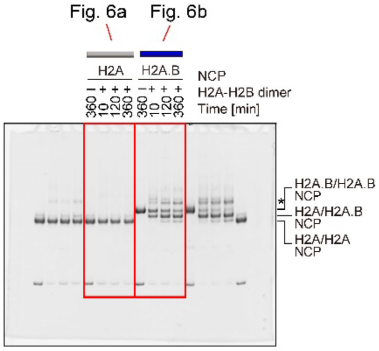


Supplementary Fig. 8

Histone exchange assay #2

Fig. 6c

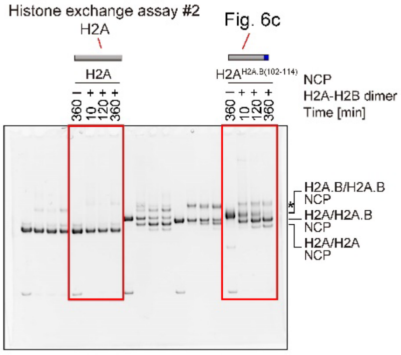

Supplementary Fig. 2a

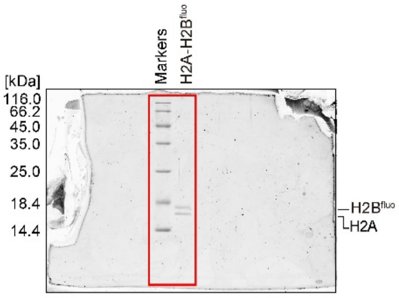


CBB

Supplementary Fig. 2b

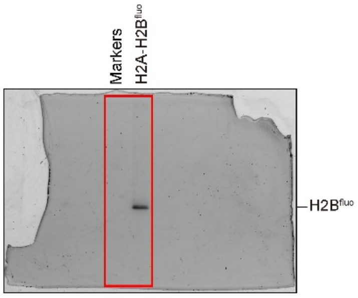


Alexa488

Supplementary Fig. 3
Histone exchange assay #2 EtBr

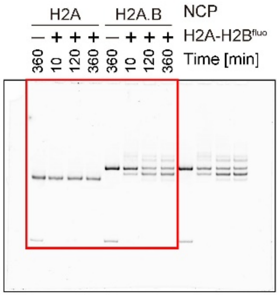


EtBr

Supplementary Fig. 3
Histone exchange assay #2 Alexa488

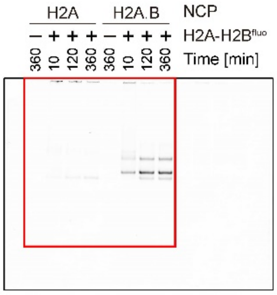


Alexa488

Supplementary Fig. 3
Histone exchange assay #2 merged

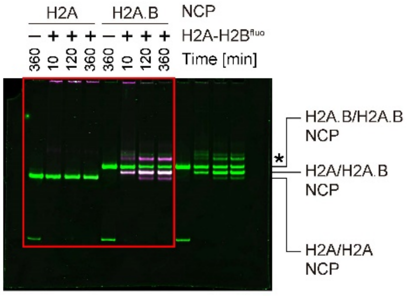


Merged

Green: EtBr
Magenta: Alexa488
NCP - histone complex

Supplementary Fig. 3
Histone exchange assay #3 EtBr

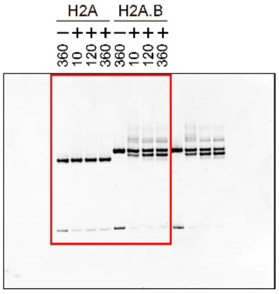


EtBr

Supplementary Fig. 3
Histone exchange assay #3 Alexa488

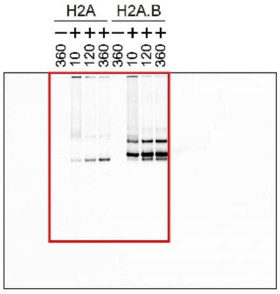


Alexa488

Supplementary Fig. 3
Histone exchange assay #3 merged

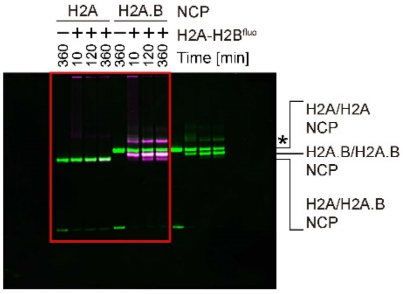


Merged

Green: EtBr
Magenta: Alexa488
NCP - histone complex

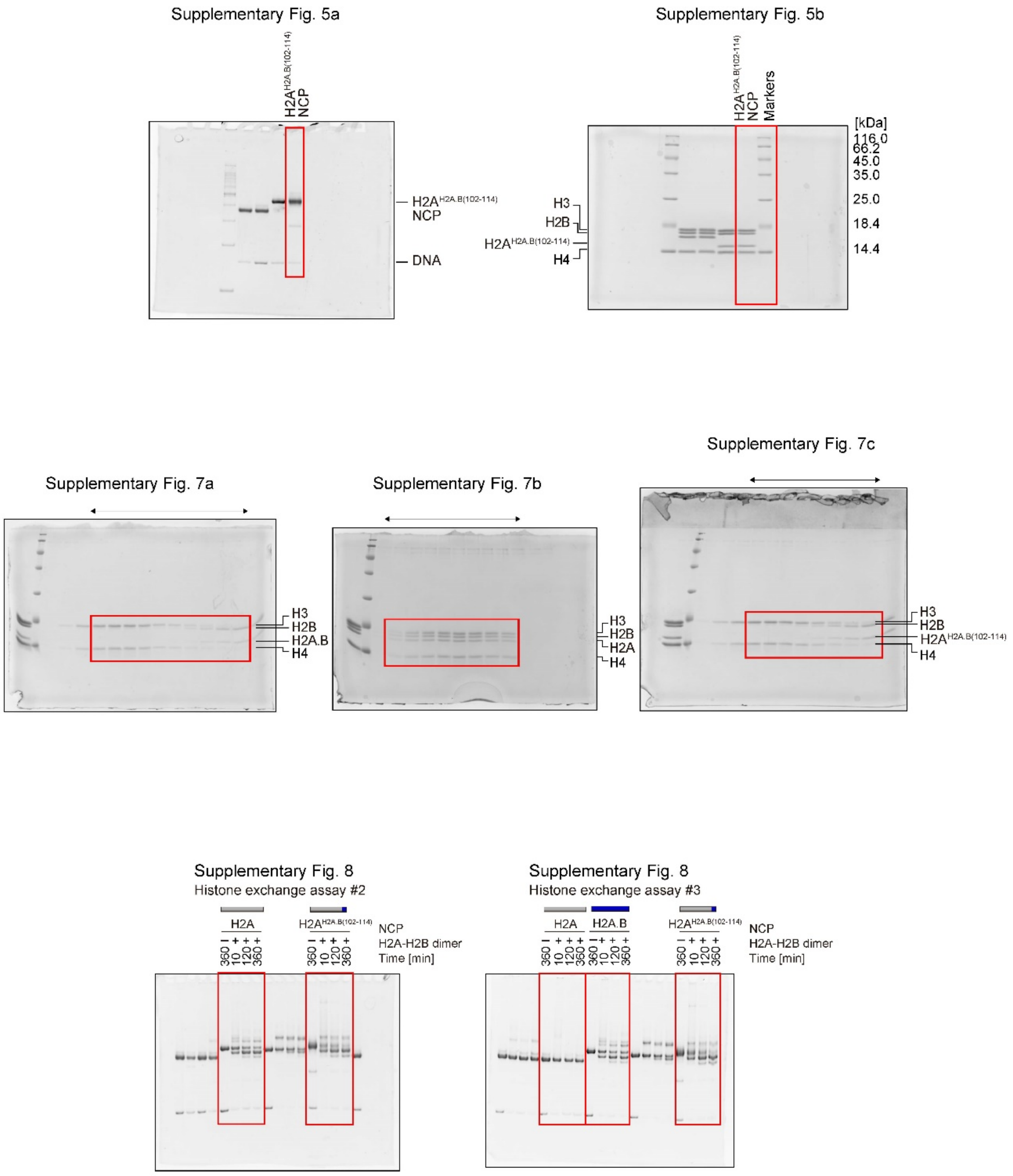


**Supplementary Figure 9. Full images of gels.**
