# Supplementary material for: Histone variant H2A.B-H2B dimers are spontaneously exchanged with canonical H2A-H2B in the nucleosome
Source: Commun Biol. 2021 Feb 12;4:191. doi: 10.1038/s42003-021-01707-z (PMC7881002; doi:10.1038/s42003-021-01707-z)
Supplement: Supplementary file 2 — Description of Additional Supplementary Files [file 42003_2021_1707_MOESM2_ESM.pdf]

Description of Additional Supplementary Files

File Name: Supplementary Data 1

Description: Data used for HS-AFM quantification in Fig. 3c and d, Fig.5c, and Supplementary Fig. 4b and c.

File Name: Supplementary Video 1

Description: HS-AFM movies of the H2A.B NCP.HS-AFM movies of three representative H2A.B NCPs on the poly-L-lysine coated mica surface, showing that the H2A.B NCP formed an open conformation during the NCP dissociation process. Pixel sizes: 112×98 $pixels^2$.

File Name: Supplementary Video 2

Description: HS-AFM movies of the H2A NCP.HS-AFM movies of three representative H2A NCPs on the poly-L-lysine coated mica surface, showing that the H2A-H2B dimers in the H2A NCP were immediately released from the disrupted NCP. Pixel sizes: 112×98 $pixels^2$.

File Name: Supplementary Video 3

Description: HS-AFM movies of the $H2A^{H2A.B(102\text{-}114)}$ NCP.HS-AFM movies of three representative $H2A^{H2A.B(102\text{-}114)}$ NCPs on the poly-L-lysine coated mica surface, showing that the $H2A^{H2A.B(102\text{-}114)}$ NCP also formed an open conformation, as observed in the H2A.B NCP. Pixel sizes: 112×98 $pixels^2$.
