# Supplementary material for: Histone variant H2A.B-H2B dimers are spontaneously exchanged with canonical H2A-H2B in the nucleosome
Source: Commun Biol. 2021 Feb 12;4:191. doi: 10.1038/s42003-021-01707-z (PMC7881002; doi:10.1038/s42003-021-01707-z)
Supplement: Supplementary file 7 — Reporting Summary [file 42003_2021_1707_MOESM7_ESM.pdf]

nature research

Corresponding author(s): Hitoshi Kurumizaka

Last updated by author(s): Dec 10, 2020

Reporting Summary

Nature Research wishes to improve the reproducibility of the work that we publish. This form provides structure for consistency and transparency in reporting. For further information on Nature Research policies, see our Editorial Policies and the Editorial Policy Checklist.

Statistics

For all statistical analyses, confirm that the following items are present in the figure legend, table legend, main text, or Methods section.

n/a	Confirmed	
[ ]	[x]	The exact sample size (*n*) for each experimental group/condition, given as a discrete number and unit of measurement
[ ]	[x]	A statement on whether measurements were taken from distinct samples or whether the same sample was measured repeatedly
[x]	[ ]	The statistical test(s) used AND whether they are one- or two-sided<br>*Only common tests should be described solely by name; describe more complex techniques in the Methods section.*
[x]	[ ]	A description of all covariates tested
[x]	[ ]	A description of any assumptions or corrections, such as tests of normality and adjustment for multiple comparisons
[x]	[ ]	A full description of the statistical parameters including central tendency (e.g. means) or other basic estimates (e.g. regression coefficient) AND variation (e.g. standard deviation) or associated estimates of uncertainty (e.g. confidence intervals)
[x]	[ ]	For null hypothesis testing, the test statistic (e.g. *F*, *t*, *r*) with confidence intervals, effect sizes, degrees of freedom and *P* value noted<br>*Give P values as exact values whenever suitable.*
[x]	[ ]	For Bayesian analysis, information on the choice of priors and Markov chain Monte Carlo settings
[x]	[ ]	For hierarchical and complex designs, identification of the appropriate level for tests and full reporting of outcomes
[x]	[ ]	Estimates of effect sizes (e.g. Cohen's *d*, Pearson's *r*), indicating how they were calculated

Our web collection on statistics for biologists contains articles on many of the points above.

Software and code

Policy information about availability of computer code

Data collection	The X-ray diffraction data were collected at the BL41XU at SPring-8 (Harima, Japan).<br>The small-Angle X-ray Scattering (SAXS) was performed with a NANOPIX instrument (RIGAKU) at the Institute of Radiation and Nuclear Science, Kyoto University.<br>The high-speed atomic force microscopy (HS-AFM) images were collected using Igor Pro Ver. 8.0.4.2. (WaveMetrics).
Data analysis	The diffraction data were scaled and processed using the HKL2000 program. The molecular replacement was performed with the PHASER program. The atomic coordinates were refined using the PHENIX and Coot programs.<br>The observed SAXS intensity was corrected for background scattering, empty cell scattering, buffer scattering, and transmission factors, and subsequently converted to the absolute scale by SAngler (http://pfwww.kek.jp/saxs/SAngler.html). The Guinier analysis was performed with the linear least square method by Igor Pro (7.04).<br>HS-AFM images were analyzed using Igor Pro Ver. 8.0.4.2. (WaveMetrics) and ImageJ.

For manuscripts utilizing custom algorithms or software that are central to the research but not yet described in published literature, software must be made available to editors and reviewers. We strongly encourage code deposition in a community repository (e.g. GitHub). See the Nature Research guidelines for submitting code & software for further information.

Data

Policy information about availability of data

All manuscripts must include a data availability statement. This statement should provide the following information, where applicable:

- Accession codes, unique identifiers, or web links for publicly available datasets
- A list of figures that have associated raw data
- A description of any restrictions on data availability

The structural data of the NCP shown in Supplementary Fig. 1 have been deposited to the Protein Data Bank (PDB ID: 6V2K).

Field-specific reporting

Please select the one below that is the best fit for your research. If you are not sure, read the appropriate sections before making your selection.

☒ Life sciences ☐ Behavioural & social sciences ☐ Ecological, evolutionary & environmental sciences

For a reference copy of the document with all sections, see nature.com/documents/nr-reporting-summary-flat.pdf

Life sciences study design

All studies must disclose on these points even when the disclosure is negative.

Sample size	Since our study did not contain statistical test, we did not perform sample-size calculation.
Data exclusions	No data were excluded from analyses.
Replication	The reproducibility for histone exchange assay was confirmed by three independent experiments. The reproducibility for gel filtration assay was confirmed by two independent experiments.
Randomization	This was not relevant to our study.
Blinding	This was not relevant to our study.

Reporting for specific materials, systems and methods

We require information from authors about some types of materials, experimental systems and methods used in many studies. Here, indicate whether each material, system or method listed is relevant to your study. If you are not sure if a list item applies to your research, read the appropriate section before selecting a response.

Materials & experimental systems

n/a	Involved in the study	
☒	☐	Antibodies
☒	☐	Eukaryotic cell lines
☒	☐	Palaeontology and archaeology
☒	☐	Animals and other organisms
☒	☐	Human research participants
☒	☐	Clinical data
☒	☐	Dual use research of concern

Methods

n/a	Involved in the study	
☒	☐	ChIP-seq
☒	☐	Flow cytometry
☒	☐	MRI-based neuroimaging
